# How to Face Chronic Liver Disease: The Sinusoidal Perspective

**DOI:** 10.3389/fmed.2017.00007

**Published:** 2017-02-10

**Authors:** Anabel Fernández-Iglesias, Jordi Gracia-Sancho

**Affiliations:** ^1^Liver Vascular Biology Research Group, Barcelona Hepatic Hemodynamic Laboratory, IDIBAPS Biomedical Research Institute – CIBEREHD, Barcelona, Spain

**Keywords:** cirrhosis, hepatic stellate cells, liver sinusoidal endothelial cells, HSC, LSEC

## Abstract

Liver microcirculation plays an essential role in the progression and aggravation of chronic liver disease. Hepatic sinusoid environment, mainly composed by hepatocytes, liver sinusoidal endothelial cells, Kupffer cells, and hepatic stellate cells, intimately cooperate to maintain global liver function and specific phenotype of each cell type. However, continuous liver injury significantly deregulates liver cells protective phenotype, leading to parenchymal and non-parenchymal dysfunction. Recent data have enlightened the molecular processes that mediate hepatic microcirculatory injury, and consequently, opened the possibility to develop new therapeutic strategies to ameliorate liver circulation and viability. The present review summarizes the main cellular components of the hepatic sinusoid, to afterward focus on non-parenchymal cells phenotype deregulation due to chronic injury, in the specific clinical context of liver cirrhosis and derived portal hypertension. Finally, we herein detail new therapies developed at the bench-side with high potential to be translated to the bedside.

## Introduction—Chronic Liver Disease (CLD)

Chronic liver disease is one of the most important causes of death worldwide representing 1.03 million deaths per year (1). A variety of toxicants may induce the initiation and progression of CLD, being excessive alcohol consumption, viral hepatitis infection, and hepatic steatosis the most predominant in our time.

**Figure 1:**
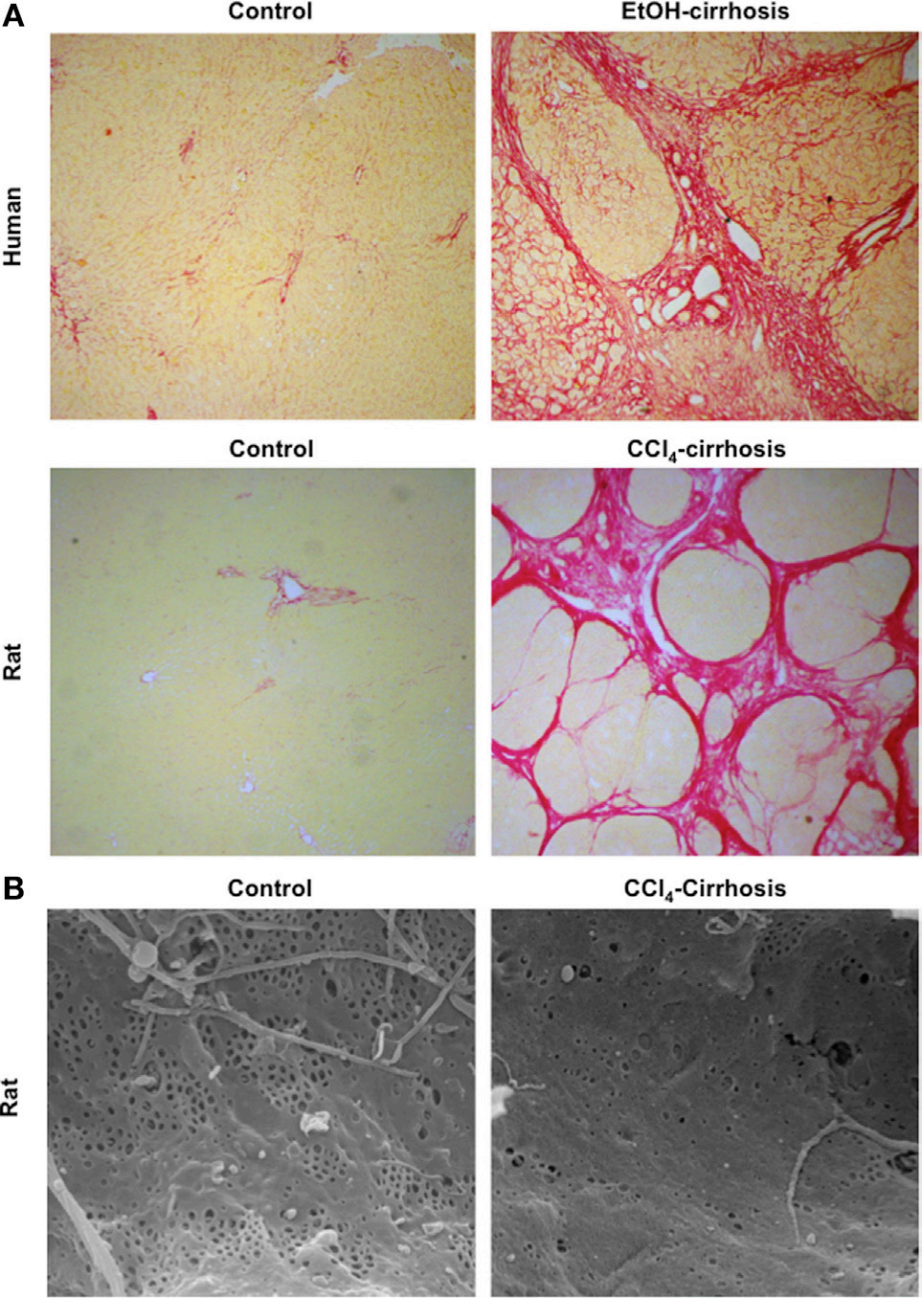
**Major morphological characteristics of microvascular sinusoidal dysfunction in chronic liver disease (CLD)**. Continuous wound-healing processes in sinusoidal cells due to exogenous toxicants lead to marked changes in their phenotype. Exaggerated production and deposition of extracellular matrix, together with parenchymal extinction and vascular occlusion, lead to profound architectural distortion [as seen in panel **(A)**: sirius red staining of control and cirrhotic human (top) and rat (bottom) livers, magnification 4×]. In addition, during CLD, liver sinusoidal endothelial cells become rapidly de-differentiated exhibiting marked reduction in their porosity, defined as fenestrae disappearance and basal membrane formation [panel **(B)**: sinusoidal fenestrae analysis using scanning electron microscopy in livers from control and cirrhotic rats, magnification 15000×].

One of the key mechanisms contributing to CLD progression is the continuous production and deposition of extracellular matrix (ECM) components such as collagen and glycoproteins, resulting in significant hepatic fibrosis and ultimately leading to the development of liver cirrhosis (or advanced CLD). Histologically cirrhosis is characterized by the formation of aberrant nodules and fibrotic septa in the parenchyma (Figure 1A) (2, 3).

Cirrhosis, as a dynamic process, can be clinically classified in different stages: (1) compensated cirrhosis without varices, (2) compensated cirrhosis with varices, (3) decompensated with ascites, and (4) decompensated with variceal bleeding. In the last stage, patients are more vulnerable to several complications such as infection, thrombosis, and development of hepatocellular carcinoma (3, 4). From a therapeutic point of view, different stages of the disease can also be considered as different “windows” for treatment; therefore, understanding the pathophysiology of CLD results essential to develop and apply effective treatments to patients with cirrhosis.

Portal hypertension (PH) is the most common and dreadful complication of CLD, and it occurs when the hepatic venous pressure gradient (HVPG) increases above 10 mmHg. Current therapies for PH aim to reduce HVPG below 12 mmHg (or 20% lower than basal pressure gradient) since it will markedly reduce the probability of decompensation and improve overall survival (5). The pathophysiology of PH defines the phenotypic alterations of all sinusoidal cells responsible for the hepatic vascular abnormalities that lead to the increment in portal pressure (6, 7). Indeed, the increase in the hepatic vascular resistance (HVR) due to both the architectural distortion, caused by deregulated fibrosis, and the microvascular dysfunction of sinusoidal cells is the primary factor in the development of PH (8–11). Secondary to HVR increment, mesenteric hypervolemia further contributes to aggravate and perpetuate the PH syndrome (12, 13).

Although CLD represents a very severe condition for humans, there are no available treatments to promote regression of liver fibrosis and the parallel amelioration of PH. In this sense, current research efforts mainly focus on developing effective therapeutic options to improve the phenotype of sinusoidal cells, which would lead to a global improvement of liver function.

## Sinusoidal Cells Under the CLD Environment

**Figure 2:**
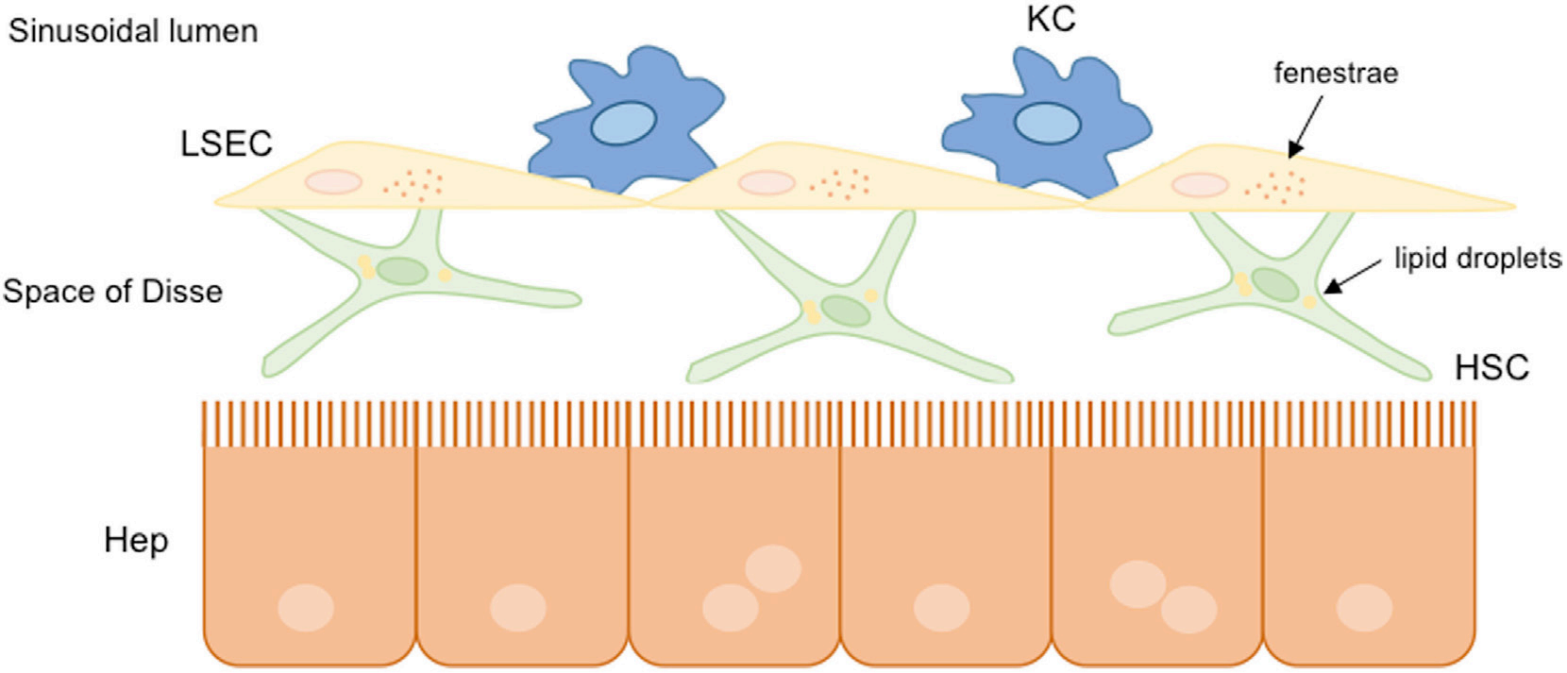
**Hepatic sinusoidal cells**. In healthy livers, parenchymal and non-parenchymal hepatic cells are well organized and communicated to each other. Hepatic stellate cells (HSC) are situated into the space of Disse between hepatocytes and liver sinusoidal endothelial cells (LSEC). Liver resident macrophages, known as Kupffer cells (KC), are situated in the hepatic sinusoid lumen. Under normal conditions, hepatic cells present a functional structure: HSC store lipid droplets, hepatocytes possess microvilli indicating functionality, and the presence of fenestrae in LSEC allows the communication with other hepatic cells.

Non-parenchymal liver cells are well organized within the hepatic sinusoidal environment. They are indeed perfectly communicated to each other to transmit different information related to physiological and pathophysiological conditions. Parenchymal cells are separated from liver sinusoidal cells by the space of Disse, where hepatic stellate cells (HSC) are located together with the components of ECM. The Kupffer cells (KC) reside in the sinusoid lumen. Liver cells are communicated with blood components through the specific fenestrae present in the liver endothelium, specifically in the cytoplasm of the liver sinusoidal endothelial cells (LSEC) (Figure 2).

During CLD progression, liver cells, and especially sinusoidal cells, try to adapt to this harmful environment changing their structure and function. These changes result in the alteration of vasodilatory, anti-inflammatory, and antioxidant programs that are essential for maintaining the healthy environment of the liver. Due to these alterations, liver sinusoidal cells switch from a protective phenotype to a pro-fibrotic one, favoring the development of cirrhosis, its main complication PH, and ultimately liver failure (6, 8). The major adaptations and changes in sinusoidal cells phenotype are described below. Please note that the multiple deregulations and adaptations that parenchymal cells undergo during CLD, which also influence non-parenchymal cells phenotype, are intentionally omitted in the present review due to length restrictions.

### Hepatic Stellate Cells

Hepatic stellate cells were described for the first time as star-shaped cells by Carl von Kupffer in 1876, although these were renamed as fat-storing cells by Toshio Ito. Afterward, Kajiro Wake identified that HSC were loaded with vitamin-A fat droplets. The characteristic of storing lipids droplets allowed characterizing HSC as low-density perisinusoidal cells.

The main functions of quiescent HSC (qHSC) remain partly unidentified, being the storage and metabolism of retinoids a major one. qHSC also regulate the sinusoidal blood flow thanks to their contractile capacity, although this function is more remarkable after their activation due to liver injury. In addition, qHSC produce several growth factors that are essential to modulate the function and proliferation of other hepatic cell types.

#### HSC Facing CLD: Activation

In response to liver injury, qHSC become “activated” (aHSC) toward an α-smooth muscle actin (α-SMA)-positive cell type characterized by enhanced contractility, increased migratory capacity, and high deposition of ECM components. In this regard, different molecular mechanisms are involved in HSC activation during CLD. These include the platelet-derived growth factor (PDGF)-α and -β receptors, the transforming growth factor β (TGFβ) signaling, and the Hedgehog (Hh) and canonical Wnt/β-catenin signaling pathways among others. See below a summary of them.

**Figure 3:**
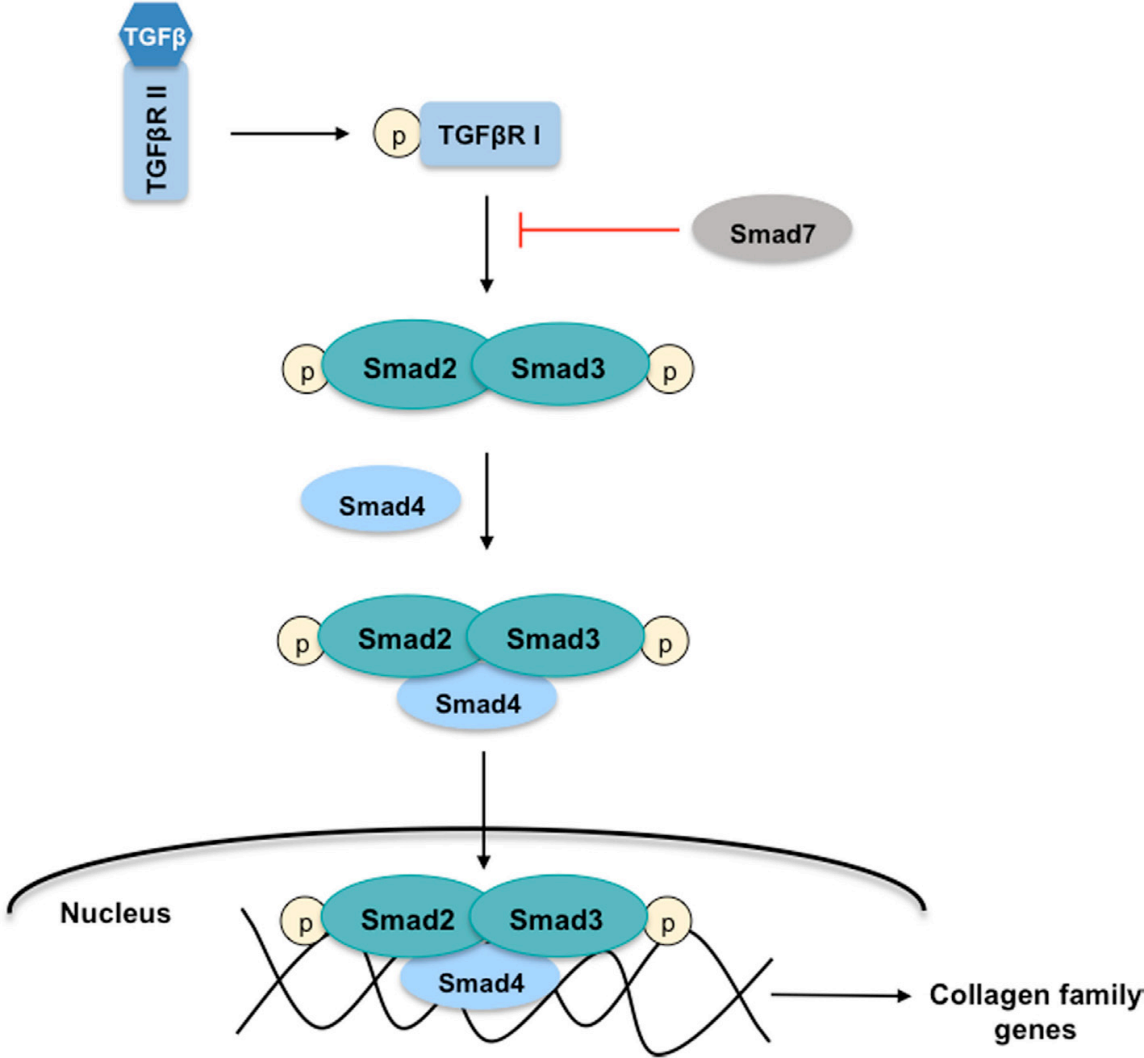
**TGFβ-Smad signaling pathway in HSC**. The cytokine-transforming growth factor β (TGFβ) interacts with the active type II receptor (TGFβRII) permitting the phosphorylation of the type I receptor (TGFβRI). The proteins Smad2 and Smad3 are recruited for the TGFβRI where both are phosphorylated. This action allows the formation of the protein-complex Smad2, Smad3, and Smad4 that is translocated into the nucleus to regulate the transcription of target genes such as the collagen family genes. On the contrary, the protein Smad7 inhibits the Smad2 and Smad3 phosphorylation.

In the initial phases of HSC transdifferentiation, PDGF-α and -β receptors are responsible for the modifications in their contractile and fibrogenic characteristics. PDGF receptors belong to the family of tyrosine kinase proteins that interact with their ligands to phosphorylate an internal tyrosine residue, activating several proliferation and migration signaling pathways, such as the Smad signaling cascade (14). It has been described that PDGF-α receptor promotes the TGFβ signaling in HSC through a Smad-dependent manner to ultimately induce differentiation of quiescent cells into myofibroblasts (15). As stated, TGFβ is one of the fibrogenic cytokines upregulated during the differentiation of HSC through the activation of Smad proteins members (Figure 3). TGFβ binds to the constitutively active type II receptor, which then recruits and phosphorylates the type I receptor. Smad2 and Smad3 proteins are phosphorylated by the interaction with type I receptor allowing the formation of a protein complex with Smad4. This complex is translocated into the nucleus where it regulates the transcription of target genes such as the collagen family genes. On the contrary, Smad7 is a TGFβ signaling inhibitory protein that inhibits the phosphorylation of Smad2 and Smad3 (12).

Moreover, TGFβ stimulates collagen transcription through a hydrogen peroxide (H_2_O_2_)- CCAAT-enhancer-binding proteins (C/EBPβ)-dependent mechanism (16, 17). Accordingly, oxidative stress plays a key role in HSC activation. Major sources of reactive oxygen species (ROS) in HSC include NADPH oxidases and dysfunctional mitochondria (18).

Other transcriptional programs contributing to HSC activation include the canonical Hh and canonical Wnt/β-catenin signaling pathways.

**Figure 4:**
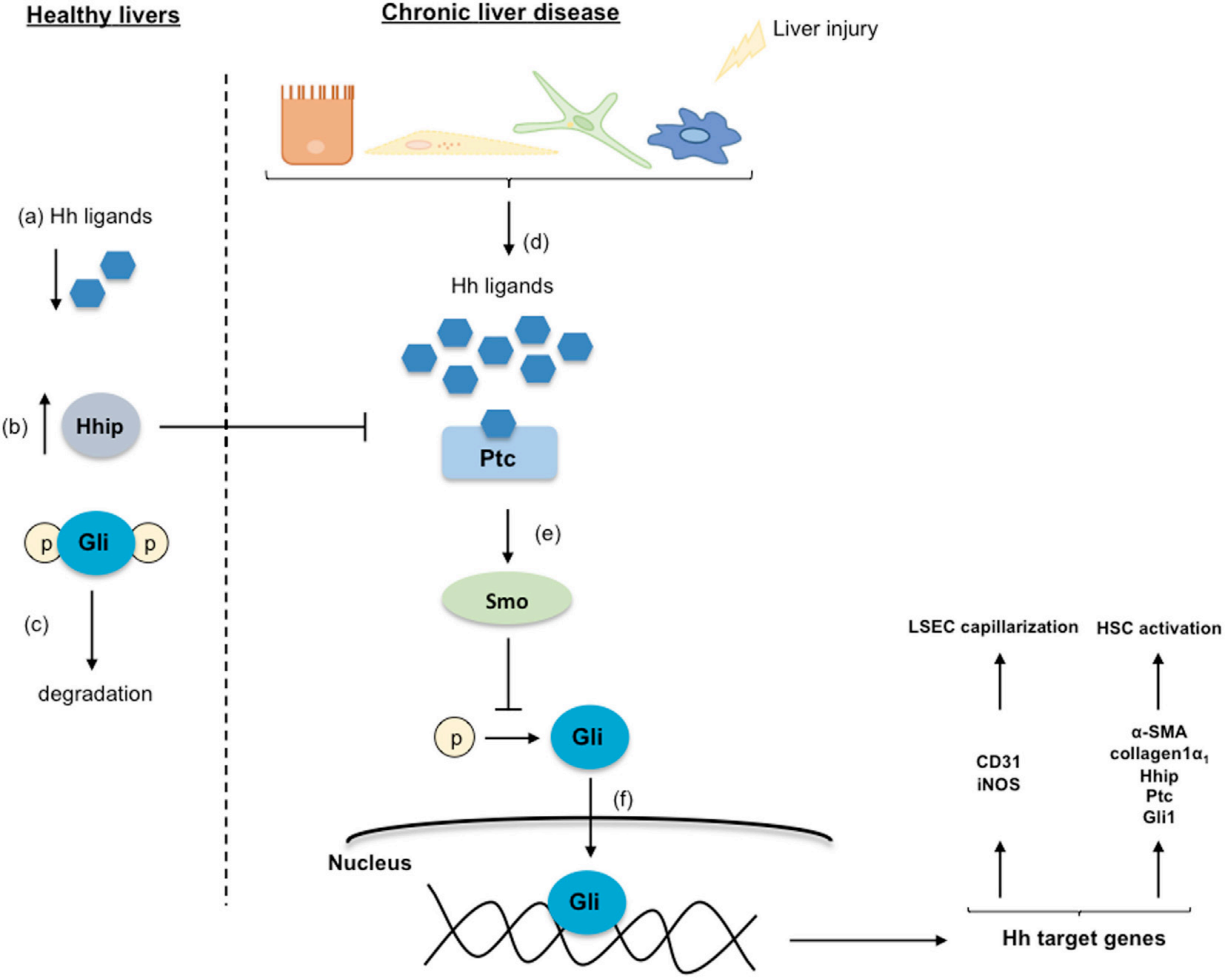
**Hedgehog signaling pathway**. In healthy livers, the Hedgehog (Hh) pathway is inactivated in response to the low-expression of the Hh ligands (a), and to the inhibition of the Hh ligands and the receptor Patched (Ptc) interaction by the over-expressed Hh-interacting protein (Hhip) (b). Thus, the Glioblastoma (Gli) proteins are phosphorylated, ubiquitinated, and degraded (c). Under chronic liver disease environment, hepatic cells are able to secrete Hh ligands (d) that through the interaction with Ptc liberate the protein Smoothened (Smo) (e). Smo inhibits Gli degradation allowing its translocation into the nucleus where regulates target genes involved in the transdifferentiation of HSC and in the liver sinusoidal endothelial cell capillarization (f).

Different studies described that Hh ligands regulate the activation of HSC promoting the transformation of qHSC to myofibroblasts, the accumulation of which contributes to CLD development (19, 20). As shown in Figure 4, in healthy livers, Hh ligands are absent or they are low expressed, and contrarily, the Hh-interacting protein (Hhip) is highly expressed disrupting the engagement between the Hh ligand and its receptor. However, when liver injury occurs, the production of Hh ligands increases, thus activating this signaling pathway. Particularly, it has been described that qHSC produce large amounts of Hhip, but upon activation, they produce Hh ligands through a PI3K/AKT-dependent mechanism (21). The Hh ligands (Sonic Hh, Indian Hh, or Desert Hh) interact with the receptor Patched (Ptc) liberating the Smoothened (Smo) molecule. Smo inhibits the degradation of the Glioblastoma transcription factors (Gli) that are translocated to the nucleus influencing the expression of target genes that play key roles in the differentiation of HSC to myofibroblasts, and stimulating their proliferation and migration (22, 23). Gli1 and Gli2 generally increase gene transcription, while Gli3 can either increase or decrease gene transcription depending on its posttranslational modification (23, 24). Indeed, the transcription factor Gli2 is implicated in myofibroblast transformation by regulating the expression of Hh pathway components (Ptc, Hhip, and Gli1) as well as key activation genes including α-SMA and collagen type 1 (25, 26). On the other hand, the Hh pathway is also essential for the communication and the crosstalk between the HSC, the parenchymal, and sinusoidal cells through the formation of exosomes that contain functional Hh proteins (27).

The Wnt family proteins stimulate the β-catenin translocation to the nucleus to modulate different proliferation and pro-inflammatory genes. Although there are some studies revealing that the activation of Wnt/β-catenin may maintain the quiescence of HSC (28, 29), growing literature supports the concept that activation of this signaling pathway contributes to HSC transdifferentiation. Moreover, β-catenin interacts with Smad proteins in the TGFβ pathway further promoting the transdifferentiation of HSC. Considering the controversial results regarding the Wnt/β-catenin molecular pathway, further studies are needed to clarify its implication in the HSC activation process (30).

### Liver Sinusoidal Endothelial Cells

Liver sinusoidal endothelial cells (LSEC) are the very specialized and unique endothelial cells that compose the vascular sinusoidal walls in the liver. LSEC form a permeable barrier due to the presence of fenestrae in their cytoplasm and the lack of organized basement membrane. Prof. Eddie Wisse described LSEC in the 70s using the electron microscopy technique.

It is important to denote that the peculiar discontinuous endothelium of the liver reduces the distance between the sinusoid and hepatocytes allowing their oxygenation and facilitating their exposition to molecules (<200 nm) from the portal circulation. Liver sinusoids receive 30% of blood from the hepatic artery (highly oxygenated) and 70% from the portal vein (low oxygen). Therefore, fenestrae of the liver endothelium are indispensable for a proper oxygen supply to all hepatic cells.

Besides their sieve function, LSEC play a key role in the regulation of the hepatic vascular tone through the production of a variety of vasoactive molecules, probably being nitric oxide (NO) the most relevant one (31). In LSEC, NO is synthesized by the endothelial nitric oxide synthase (eNOS), which is transcriptionally regulated by the transcription factor Kruppel-like factor 2 (KLF2). In fact, KLF2 is highly expressed in the endothelium conferring vasoprotection by the induction of different vasodilator and anti-inflammatory genes (32).

#### LSEC Facing CLD: Capillarization

**Figure 5:**
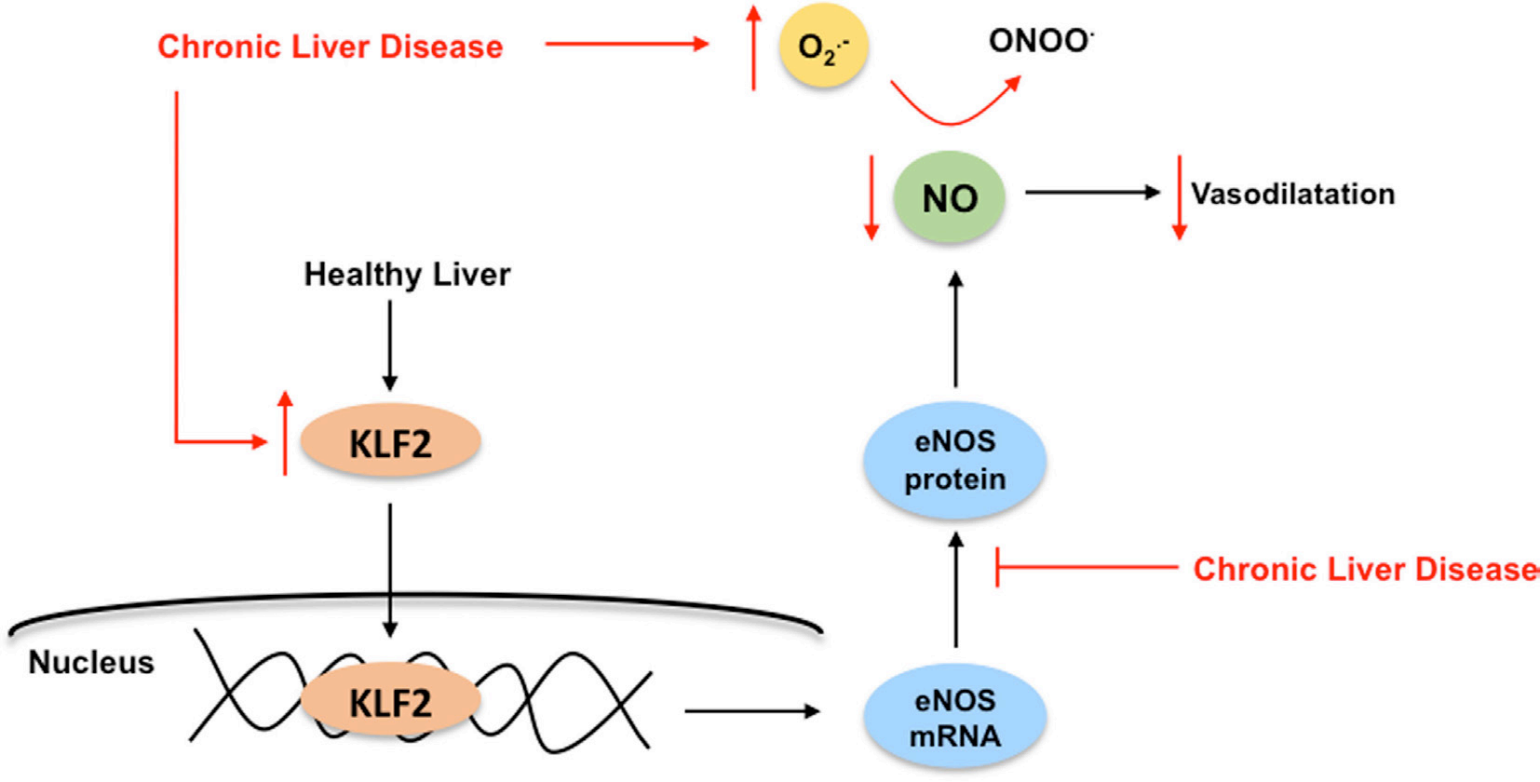
**Kruppel-like factor 2 (KLF2) regulates nitric oxide (NO) bioavailability in liver sinusoidal endothelial cells**. In healthy livers (black arrows), the transcription factor KLF2 regulates the endothelial nitric oxide synthase (eNOS) expression, which is responsible for NO synthesis, ultimately exerting its vasodilatory effects in the sinusoid. Under chronic liver disease environment (red arrows), and as compensatory mechanism, KLF2 is upregulated in the endothelium leading to increased eNOS mRNA expression, but not elevated eNOS protein. Moreover, elevated NO-scavenging by high levels of reactive oxygen species (specially, superoxide, ${\text{O}}_{2}^{-}$) within the cirrhotic liver reduces even more NO bioavailability. Altogether, enhancing liver microvasculature constriction.

In front of CLD, LSEC become profoundly deregulated, probably being the first hepatic cell type modifying its phenotype due to the external injury. The capillarization term refers to the loss of the specialized and unique phenotype of the LSEC in the liver. It occurs in response to liver damage, and LSEC lose their fenestrae (Figure 1B), develop basal membrane, and acquire vasoconstrictor, pro-inflammatory, and pro-thrombotic properties (8). Indeed, and as stated above, in healthy livers, LSEC regulate hepatic vascular tone promoting the proper synthesis of vasoactive molecules including vasodilators such as nitric oxide (NO), prostacyclin (PGI_2_) or carbon monoxide (CO), and vasoconstrictors-like thromboxane A2 (TXA_2_), endothelin-1 (ET-1) or leukotrienes. However, upon chronic injury, LSEC exhibit a deep imbalance favoring sinusoidal vasoconstriction, and, therefore, increasing the HVR (33, 34). Multiple mechanisms explain the imbalance in endothelial-derived vasoactive molecules. Briefly, elevated amounts of vasoconstrictors derived from increased expression of both intermediaries and key enzymes responsible for endothelin-1 or thromboxane production. In addition, low NO bioavailability is consequence of low eNOS activity and high NO-scavenging by the elevated oxidative stress present in the cirrhotic liver (35). Interestingly, it has been demonstrated that, in cirrhotic livers, KLF2 is upregulated in LSEC, which leads to increased eNOS mRNA expression, but not to elevated eNOS protein, thus suggesting a defect in its translation or degradation (32) (Figure 5).

It has been described that the Hh signaling pathway contributes to the capillarization of LSEC. The expression of Hh ligands (specifically SHh) and several Hh signaling components and target genes (Ptc receptor, Smo protein, and Gli2 and Gli3) are increased during capillarization of rat primary LSEC leading to the loss of fenestration (36) (Figure 3). Although LSEC produce Hh ligands, it remains unclear if the Hh-mediated loss in fenestrae is produced only by the direct action of the LSEC-Hh ligands that activate the Hh signaling cascade, or in addition by paracrine interactions from the Hh ligands produced by hepatocytes, HSC, cholangiocytes, and KC. In this sense, it has been shown that HSC release microparticles containing active forms of Hh ligands (SHh and IHh) that paracrinally induce changes in the expression of capillarization markers in LSEC (37).

On the contrary, vascular endothelial growth factor (VEGF) is necessary for the formation and maintenance of LSEC phenotype. Its expression depends on Hh signaling pathway and also on NO availability. Therefore, in cirrhosis, the activation of the Hh signaling pathway stimulates VEGF production, which will ultimately require autocrine production of NO by LSEC to preserve their fenestration (38, 39).

### Kupffer Cells

The liver resident macrophages, known as KC, are situated into the sinusoidal lumen, which as stated above is composed of LSEC. KC represents the 80% of the total macrophages in the body and, in the liver, they are able to renew from resident stem cells. Since KC are exposed to blood as well as to antigens and bacterial endotoxins, they constitute the primary line of defense to maintain the immune system in the liver and to provide protection secreting anti-inflammatory molecules, such as interleukin-10 (IL-10) (40).

#### KC Facing CLD: Polarization

During CLD progression, inflammation has an important role modifying the phenotype of all sinusoidal cells, being KC major players in this pathological process. Find herein an outline of the inflammatory molecular mechanisms affecting/mediated by KC.

**Figure 6:**
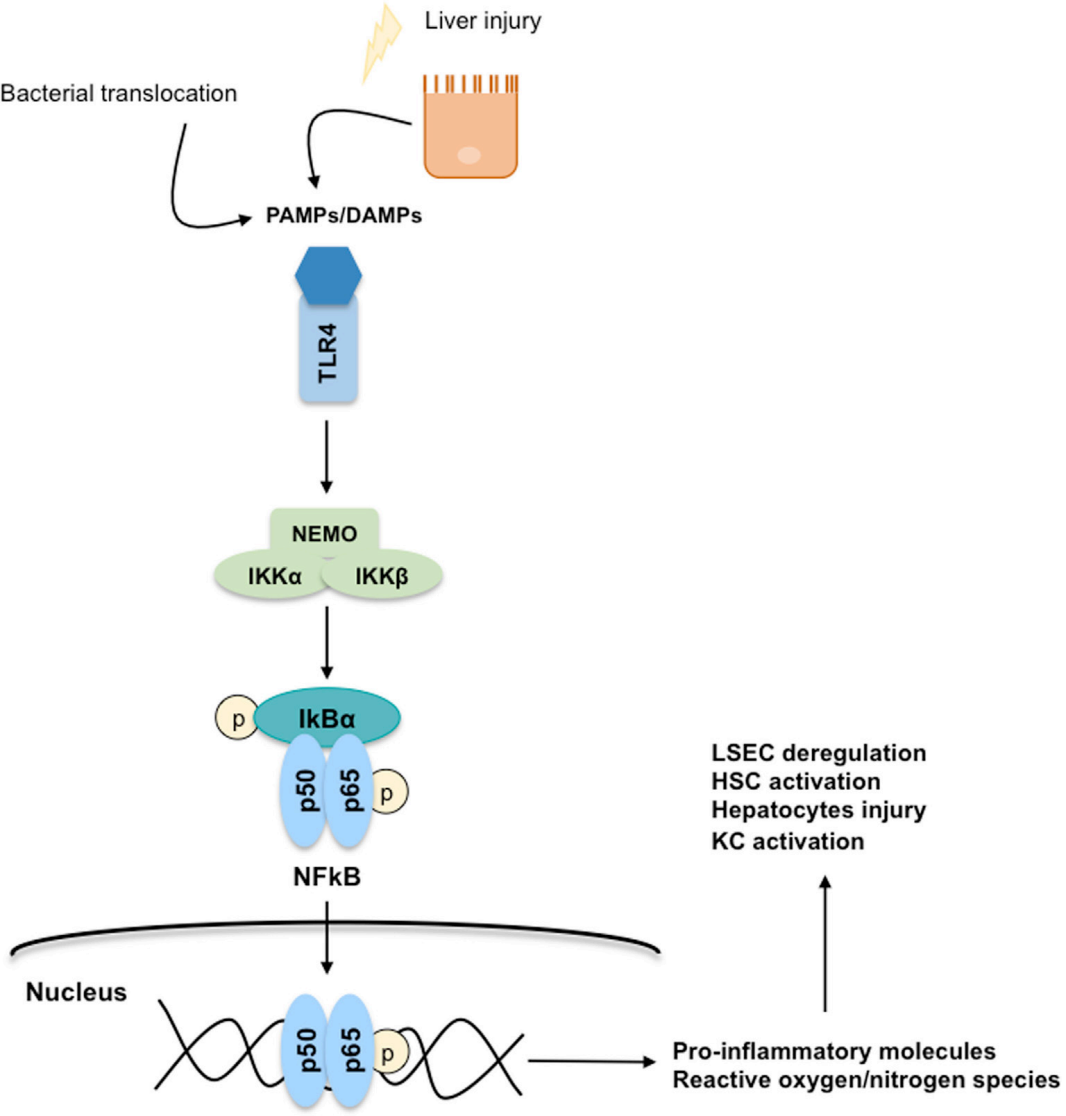
**Toll-like receptor 4 (TLR4)-NFκB signaling pathway**. The secretion of damage-associated-molecular-patterns (DAMPs) and pathogen-associated-molecular-patterns (PAMPs) from injured hepatocytes and bacterial translocation due to chronic liver injury stimulates inflammatory processes through a TLR4-NFκB mechanism predominantly on Kupffer cells. TLR4 stimulation activates the canonical NFκB signaling pathway: the subunit IκB is phosphorylated by the NEMO-complex liberating the p50–p65 NFκB dimer. Then, the NFκB dimer is translocated to the nucleus where it binds to RNA response elements to promote the transcription of pro-inflammatory cytokines, chemokines, reactive oxygen species, and reactive nitrogen species contributing to global liver injury.

During liver injury, parenchymal cells undergo apoptosis, and/or necropotosis, secreting damage-associated-molecular-patterns (DAMPs) that interact with KC through a toll-like receptor 4 (TLR4)-mediated mechanism. Importantly, in CLD, KC-TLR4 is also activated by pathogen-associated-molecular-patterns (PAMPs) produced by the bacteria translocated from the gut to the liver, or by the gut-bacterial products reaching the liver due to gut leakiness (41). Regardless of the mechanism, TLR4 stimulation directly activates the canonical NFκB signaling pathway (42, 43) involving the phosphorylation of the subunit IκB by the NEMO-complex leading to the translocation of the p50–p65 NFκB dimer to the nucleus (Figure 6). In the nucleus, NFκB binds to RNA response elements to promote the transcription of several pro-inflammatory cytokines (TNFα, IL-6, and TGFβ), chemokines, and reactive oxygen and nitrogen species. These pro-inflammatory molecules further contribute to global liver injury through paracrine communications. Indeed, they promote hepatocyte injury, deregulation of LSEC, and activation of HSC. Interestingly, it has also been reported that TNFα regulates the apoptotic agents bcl-xL and p53, altogether promoting the survival of rat-activated HSC (33).

Chemokine family comprises a total of 70 proteins including chemokine ligands and chemokine receptors, and as stated above, are involved in the progression of liver fibrosis. The chemokine CCL2, also known as MCP-1, is an important chemokine implicated in the initiation of the inflammation process, in fibrogenesis, and in the development of NAFLD and NASH (44). It is secreted by KC, also by injured hepatocytes and activated HSC, with the subsequent accumulation of its receptor CCR2 in inflammatory monocytes. In fact, the CCL2–CCR2 complex is associated with the accumulation of macrophages within the liver altogether contributing to hepatic damage and HSC activation (45). Another well-characterized chemokine implicated in the progression of liver fibrosis is CCL5 or RANTES (46). Through the interaction with its CCR5 receptor, CCL5 is directly implicated in the fibrogenic response mainly driven by HSC. On the contrary, the chemokine CX3CL1 exerts anti-inflammatory and antifibrogenic effects. Its receptor CX3CR1 represents a fibrosis resolution option mediating the restoration of macrophage phenotype by the inhibition of CCL2–CCR2 effects (47).

Finally, activated KC play also a prominent role regulating the hepatic vascular tone in CLD. Indeed, it has been demonstrated that liver resident macrophages release substantial amounts of vasoconstrictor molecules, including TXA_2_ and cysteinyl-leukotrienes, that acting of neighboring HSC increase HVR (48, 49).

## Taking Care of Sinusoidal Cells: Novel Treatments for CLD and PH

As described in the introduction, and although CLD and its complications represent a serious health problem in our society, there are not available treatments to promote cirrhosis regression. From studies using the new generation of antiviral drugs, we now know that abrogation of the toxicant effector promotes some degree of spontaneous regression of fibrosis (50, 51), which can also be observed using preclinical models of cirrhosis (52). Nevertheless, additional efforts are required to find safe and reliable therapeutic options for patients with CLD and the associated clinical complications.

Considering the above exposed sinusoidal perspective, novel therapies to ameliorate CLD and its main complication (portal hypertension) should focus on improving the phenotype of all sinusoidal cells (53). Following this rationale, statins may represent a good option. Indeed, statins benefit both KC, HSC and LSEC. It has been described that statins, specifically simvastatin, are able to increase NO bioavailability in the sinusoidal compartment by increasing the transcription factor KLF2, which induces the expression of eNOS and consequently increases the availability of this vasodilator, ultimately resulting in a decrease in the hepatic vascular tone. In addition, different studies have shown that statins promote the deactivation and apoptosis of activated HSC, thus reducing collagen synthesis and ameliorating sinusoidal vasoconstriction (54–58). Importantly, HSC improvement is also KLF2-RhoK-mediated. Moreover, and by a mechanism not totally understood, statins improve the phenotype of KC, which acquire an M2-type phenotype (58). In addition to the direct cellular effects of statins, we demonstrated that improvement of a specific sinusoidal cell type leads to concomitant improvement of neighboring cells through a paracrine mechanism (8).

Work from our team using aged animals suffering CLD (a preclinical model that closer resembles the clinical reality) reinforces the concept of global sinusoidal improvement in response to statins. Indeed, semi-chronic administration of simvastatin promotes fibrosis regression and marked amelioration of PH (59), thus encouraging its use at the bedside. In this regard, different clinical trials and observational studies already proposed the benefits of statins in patients suffering CLD (60–62). Although out of the scope of the present study, careful analysis of such excellent studies suggest that a specific “statins therapeutic window” may exist for patients with CLD. Future studies will hopefully clarify the appropriate use of these sinusoidal-protective drugs to promote regression of CLD.

Regarding the depleted NO bioavailability of cirrhotic livers, the use of antioxidants would be a good strategy to reduce oxidative stress, particularly superoxide anion (${\text{O}}_{2}^{-}$), which reacting with NO leads to the formation of peroxynitrite anion (ONOO^−^) and, therefore, results in reduced NO availability (63). Additionally, ROS directly react with eNOS inhibiting its phosphorylation and increasing its inhibitors (64). Previous studies tested several natural products with antioxidants properties such as vitamin C, flavonoids, and resveratrol in clinical and preclinical models (65–67), with mixed results in terms of improvement of CLD.

Additionally, several literature have defined different natural compounds, such as curcumin and tea polyphenols (EGCG and theaflavin), as inhibitors of the Wnt/β-catenin pathway resulting in HSC deactivation and amelioration of liver fibrosis (68), as well as regulating different cellular pathways implicated in liver carcinogenesis (69). New therapeutic strategies focused on the Wnt/β-catenin signaling pathway would be an interesting strategy to ameliorate hepatic fibrosis; however, this pathway also plays an important role in liver regeneration and hepatic zonation (70–72).

Several strategies aimed at improving inflammation and derived monocyte infiltration have been developed (45). It has been reported in preclinical models of fibrosis that inhibition of CCL2, or its receptor CCR2, promotes marked reduction in hepatic fibrosis (73). Additionally, the use of a CCL5 antagonist ameliorates liver fibrosis in mice (46). On the other hand, it is conceivable that the activation of CX3CL1 or the receptor CX3CR1 would be an alternative option to promote HSC deactivation and regression of liver fibrosis (47). As mentioned before, these chemokines are regulated by the NFκB transcription factor. However, the effects of modulating this molecular pathway on hepatic fibrosis is not clear nowadays; under physiological conditions, the activation of NFκB is essential to prevent hepatocytes apoptosis, being, therefore, protective, but an over-activation results in hepatic inflammation (74). Nevertheless, different preclinical studies have demonstrated that inhibition of NFκB accelerates the regression of hepatic fibrosis (42, 43). Similarly, the use of TLR4 antagonists represents an attractive option to ameliorate the inflammation processes occurring in the cirrhotic liver (41).

Many other therapeutic options are being characterized, both at benchside and bedside, with promising preliminary results. These include pan-caspase inhibitors (75, 76), GLP-1R and FXR agonists (77–79), and lysyl oxidase inhibitors (80). Upcoming results will clarify their usefulness and applicability in patients with CLD, nevertheless, their success will certainly rely on their capability to efficiently improve the phenotype of sinusoidal (and parenchymal) cells. Hopefully, future therapeutic developments will derive from synergistic efforts from basic researchers (with large knowledge on the biology of sinusoidal cells), clinicians (with deep experience in CLD pathophysiology), and private entities (aimed at improving patients health through supporting preclinical research). Concluding that the chances to succeed in the battle against CLD are directly proportional to the real improvement in the phenotype of hepatic cells considering the pathophysiology of the disease: the way to go on!

## Author Contributions

AF-I: conception of the work, drafting the manuscript, and preparation of figures. JG-S: conception of the work, critical revision of manuscript and figures. Both authors approved the final version of the article.

## Conflict of Interest Statement

The authors declare that the research was conducted in the absence of any commercial or financial relationships that could be construed as a potential conflict of interest.
